# Infantile Hemangioma: A Common Lesion in a Vulnerable Population

**DOI:** 10.3390/ijerph20085585

**Published:** 2023-04-20

**Authors:** Samon Nazemian, Shohreh Sharif, Esther L. B. Childers

**Affiliations:** 1 Pediatric Dentistry Resident Department of Pediatric Dentistry, College of Dentistry Howard University, Washington, DC 20059, USA; 2 Greater Washington Dentistry, 3700 Joseph Siewick Drive, Fairfax, VA 22033, USA; 3 Department Oral and Maxillofacial Pathology, College of Dentistry Howard University, Washington, DC 20059, USA

**Keywords:** hemangioma, infant, dentistry, oral, neoplasm, treatment, decision tree

## Abstract

Infantile hemangioma (IH) is important to all dentists, particularly dentists providing services to pediatric populations, because significant morbidity and mortality can occur from vascular lesions in children. Specialists of the oral cavity have the responsibility to identify patients with IH, a lesion that could be potentially life threatening. We present a case of IH and will provide a narrative review of the most recent literature. We discuss the diagnosis, risk stratification, treatment, complications, and impact on routine dental procedures. A proper diagnosis is crucial as oral and perioral IH are considered high-risk due to the increased risk of ulceration and feeding impairment. Referral to a hemangioma specialist for comprehensive team treatment is optimal. The natural history of IH consists of a long proliferative phase, which manifests as clinically visible growth. As a result of early encounters with patients, the pediatric dentist may often be considered the primary care provider.

## 1. Introduction

Infantile hemangioma (IH) is important to all dentists, particularly pediatric dentists, because injury can occur from this type of vascular lesion in the oral region, even during trivial trauma such as feeding. Bleeding caused by iatrogenic or minor manipulations during treatment or examination could cause bleeding and possibly morbidity or even mortality. Recognition of vascular tumors such as IH is crucial because they are the most common tumor of infancy [1]. While IH can be life threatening, the majority of these lesions resolve on their own [2]. The difficulty lies in the fact that there is no reliable method for determining which lesions will spontaneously resolve and which lesions will need intervention. Therefore, the referral to an expert is crucial for appropriate care for this vulnerable population. 

The differentiation of IH from a vascular malformation is crucial for accurate diagnosis and treatment. A vascular malformation is present at birth and grows in proportion to the infant. In contrast, IH is absent at birth but may appear and proliferate rapidly for months [3]. IH appears during the neonatal period, approximately two weeks after delivery [4]. Females are three times more affected by this vascular lesion. IH can range in size from 0.5–5.0 cm. A distinguishing characteristic of IH is the early, rapid proliferation during the neonatal period and continuing through infancy. The lesion may reach its greatest dimension of growth by the child’s 5th month of life [5]. This is followed by a phase where growth levels off. Then, most lesions slowly involute or shrink in size [1]. The lesions can either be superficial or deep. The superficial lesion is brighter red in color [6]. Deeper lesions have a blueish hue. IH are located on the head and neck most of the time [7]. The remainder of lesions are commonly located on the trunk and extremities. 

IH is a vascular tumor, which are benign neoplasms that are a result of proliferating endothelial cells. Local complications may include problems such as bleeding, tissue destruction, and pain. Systemic sequelae include thrombocytopenia, congestive heart failure (CHF), and death. Infantile hemangiomas are the most common type of benign vascular tumor, making up approximately 5–10% of all vascular tumors [8]. The lesion is more common in females than in males. White subjects presented with the lesions more than non-whites. Twins, preterm infants, advanced maternal age, and placental anomalies were associated with an increased incidence of IH. Some evidence exists that some infantile hemangiomas are inherited, but there is still not enough information to explain the inheritance model. However, the Utah Population Database reported a two-fold increase in the risk ratio for hemangiomas among siblings of an affected proband. Some studies have reported genetic variants associated with germline mutations in the VEGFR2, VEGFR3, and TEM8 genes. These genes regulate major angiogenesis-signaling pathways, suggesting hyperactivation of VEGFR2 signaling in the pathogenesis of infantile hemangiomas [9]. In another study, the expression of the GLUT 1 gene was present in infantile hemangiomas but not in other vascular tumors. Identification of genetic differences may be useful in the future [7].

The focus of this paper is to provide the reader with a case report for illustration of the condition and its management. Diagnosis and collaboration allow for successful management of patients with infantile hemangiomas by the dentist. 

## 2. Case Presentation/Materials and Methods

At an older sibling’s appointment, the patient’s mother communicated a concern about her infant, and she had the baby present with her at the time. The parent’s chief concern was, “She has a red thing that has been growing in her mouth”. The mother made an appointment for the review of the lesion, and the 5-month-old female presented to the pediatric dentist for evaluation. Her medical history was unremarkable, and she was born at full term with no complications. The lesion was not noted at birth.

The mother stated that the patient was not displaying any abnormal signs or symptoms for a child of that age. She fed regularly and had normal sleep patterns. She did not show excessive irritability. There was no history of excessive vomiting, diarrhea, fever, or cough. The mother stated she noticed the lesion was growing, and she did not remember if there had been a diagnosis, although she had inquired of the child’s pediatrician. She was concerned about the location being in the mouth and what the repercussions would be. 

**Figure 1:**
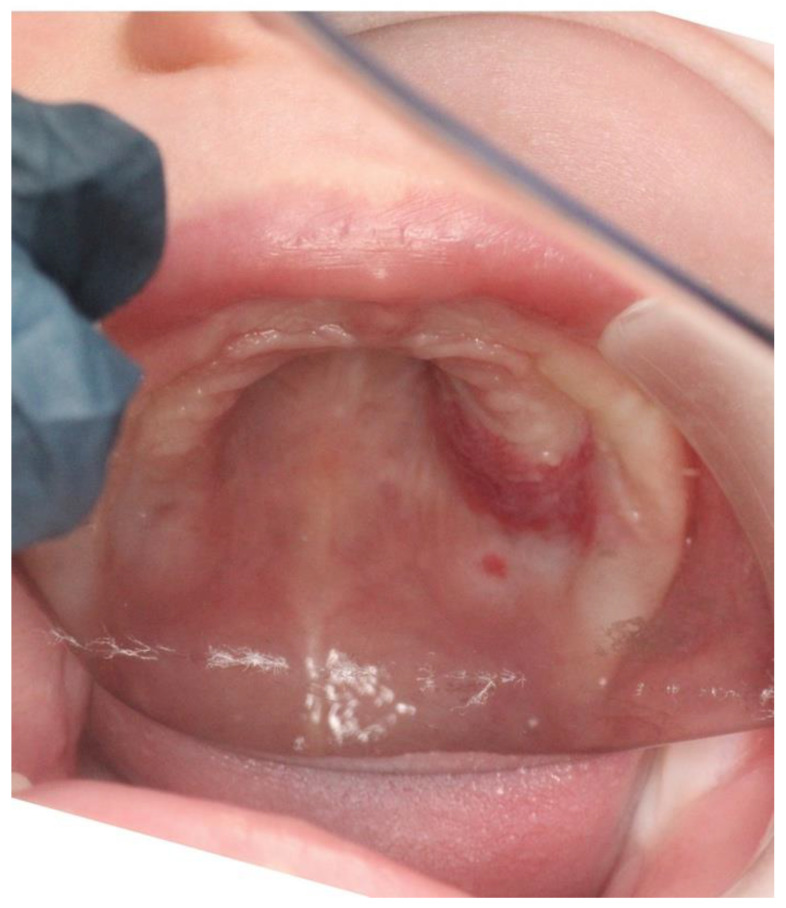
Intraoral photograph showing a 5-month-old Caucasian female with a 2.5 cm × 3.5 cm well-circumscribed, non-contiguous, red/crimson macule with a 1 mm white dot at the center. It is located on the posterior portion of the upper left alveolar ridge, spanning to the mid-palate.

**Figure 2:**
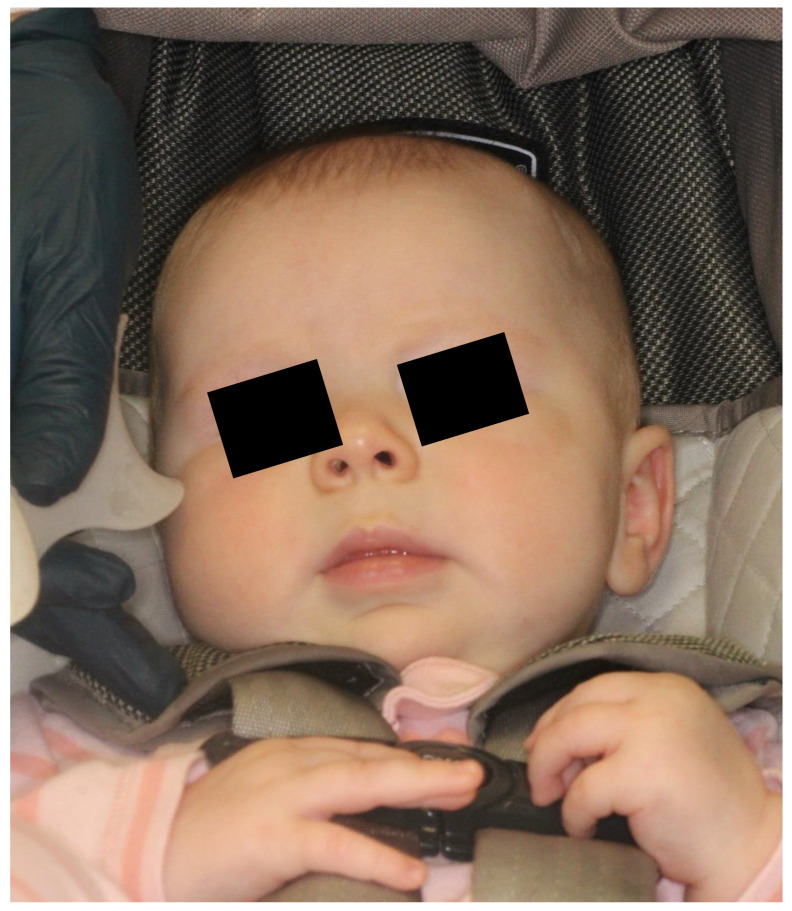
Extraoral photograph showing a well-developed 5-month-old Caucasian female that presented with the described intraoral lesion. There are no facial lesions.

**Figure 3:**
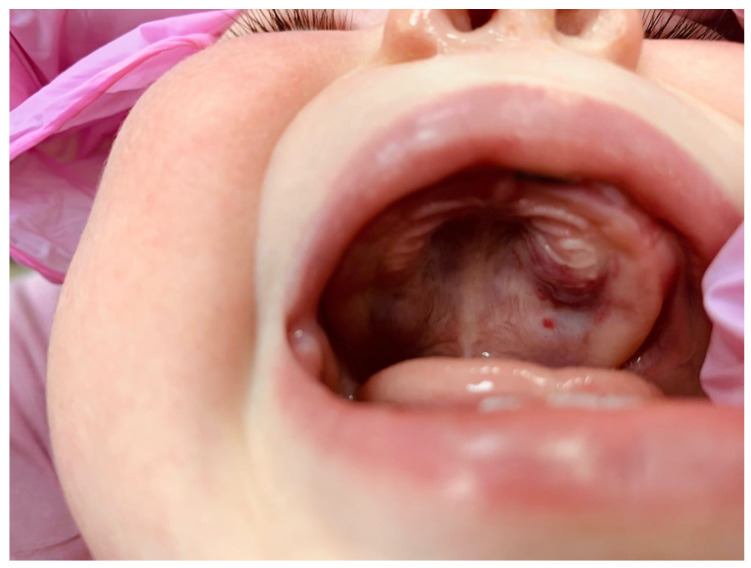
Intraoral photograph showing a 11-month-old Caucasian female with a 3.5 cm × 3.5 cm well circumscribed, non-contiguous, red/crimson macule with a 1 mm white dot at the center. It is located on the posterior portion of the upper left alveolar ridge, spanning to the mid-palate, and is anterior to the smaller red macule.

The intra-oral clinical exam showed (Figure 1 and Figure 2) a 2.5 cm × 3.5 cm well-circumscribed red/crimson macule with a 1 mm white dot in the center, located on the posterior portion of the upper left alveolar ridge spanning to the mid-palate. There were no other abnormal soft tissue findings. No radiographs were taken. A history from the parent revealed that the lesion had been present shortly after birth and had been growing since. The patient did not present with any extra-oral swelling or other oral pathology. At present, aged 11 months, the lesion has not changed size (Figure 3). 

The differential diagnosis included hemangioma, vascular malformation, or telangiectasia lesions, and a consultation with an oral and maxillofacial pathologist was conducted. The patient was placed on a monthly recall for observational appointments with a provisional diagnosis of IH. They were also referred to dermatology and ENT for further evaluation. The patient, since their initial appointment with the pediatric dentist, had visited the ENT and the dermatologist. Both of those specialists agreed that the lesion was an infantile hemangioma and are following the patient. 

## 3. Discussion

IH is the most common vascular tumor of infancy. A distinguishing characteristic is its growth cycle, usually obtained from the patient’s history. The lesions typically appear during the neonatal period, two weeks after delivery [4]. The first step of its growth cycle is early and rapid proliferation. The proliferative phase can last up to one year [10]. The lesion has been reported to reach 80% of its final size during the first 5 months of the child’s life [5]. During the proliferative phase, the skin or mucosa becomes irregular, raised, and crimson. The lesion is “high flow” during the proliferative period [3]. This means that there may be an arterial component and that there may therefore be a higher propensity for bleeding. This is followed by a plateau phase where the lesion slowly continues to grow for 1–8 years. The final stage of the life cycle is the involution phase. The color fades, and the mucosa is more pink. IH has been reported to heal both with and without scarring, telangiectasia, yellowish hypoelastic patches, and dermal atrophy [7]. Currently, features that predict prognosis are not well defined. 

Our patient’s history, as described by her mother, is consistent with the initiation and proliferation phases of IH. at our patient’s age of 5 months at initial observation, it was most likely at the end of the proliferation phase and entering the plateau. The proliferation phase could continue up until the first year of life. 

IH lesions are typically 0.05–5.0 cm in size and often affect the head and neck area [7]. They can also commonly affect the trunk and extremities. Females are three times more affected by the lesion. IH are either defined as superficial, deep, or mixed. A superficial lesion is bright in color, and a deeper lesion leaves the skin with a blueish hue [6]. The lesions are classified according to their clinical appearance as focal, intermediate, or segmental [4]. Intermediate lesions are classified as such because their features fall between the criteria for focal and segmental. A multifocal lesion consists of 10 or more lesions [4]. A majority of cases are solitary. Our patient showed a non-contiguous lesion that consisted of a larger lesion on the palate and a second, smaller one bucco-lingually across the upper left alveolar ridge.

The diagnosis of IH is based on the findings of the clinical exam and screening questions for history and duration. Most cases can be diagnosed based on growth history and appearance. Diascopy may be a useful screening tool, although caution must be used to avoid glass or plastic breakage that could induce bleeding [3]. Sometimes MRI, sonography, or other advanced imaging techniques are useful. Advanced imaging assists in defining the anatomic extent of the lesion, and vascular studies may identify a feeder vessel. This information is vital for the clinician to establish whether the lesion is limited to the subcutaneous tissues or has deeper muscular involvement. Clinical observation of IH is useful to evaluate the size, borders, color, and location of the lesion. Physical examination may determine subcutaneous or muscular tissue involvement for cutaneous locations [3]. Screening questions allow the clinician to establish the history because the time of manifestation of the lesion is helpful in distinguishing IH from other lesions in the differential.

Hemangiomas are true neoplasms characterized by proliferation and increased rates of endothelial cell turnover. Infantile hemangiomas typically develop during the first 2 months of life and demonstrate rapid proliferation between 6 and 12 months of age [7]. These can range from an erythematous macule to a deeper mass with a surface of red or blue [3]. In contrast, vascular malformations are localized anomalies due to defects in vascular morphogenesis with normal rates of cell turnover [3]. They can be capillary, venous, lymphatic, or arteriovenous in formation. Vascular malformation lesions are present at birth. They do not have the same proliferative pattern as IH; rather, they grow more proportionally to the growth of the child. Telangiectasias are harmless, small, dilated blood vessels on the skin or mucosa that can occur [11]. Capillary malformations that are visible as telangiectasias are commonly seen in newborns. These lesions fade and may be known as ‘fading macular stains’ While lesions that appear on the nape of the neck are sometimes called ‘stork bites’ and those on the glabellar region are referred to as ‘angel’s kiss’ [12].

The complications of IH can be life-threatening. Heart failure and respiratory distress have been associated [7]. Depending on the location, compression of the periorbital structures or the airway can occur. Lesions located intra-orally are at higher risk of life-threatening bleeding. Sequelae of IH include ulceration and fissure formation. Ulceration is the most common, reported to occur 16% of the time [13].

When deciding how to manage patients who present with this lesion, treatment considerations may be divided into non-active and immediate. Non-active refers to watchful waiting if the IH is less than 2 cm and specifically located on the trunk or extremities. During watchful waiting, the patient is followed for sequential appointments for close monitoring [13]. Chronological photographs help document the biologic behavior of the lesion. During this time, the proper referrals, such as oral and maxillofacial pathology, oral and maxillofacial surgery, ENT, or dermatology, are appropriate for dentists or other clinicians. Lesions that are located on the face or in the oral cavity fall under the high and highest risk categories [13], regardless of size. Oral and perioral lesions may become ulcerated during feeding or from trivial trauma associated with mastication. Oral or perioral lesions fall under the high-risk category because ulceration can lead to excessive bleeding or impaired feeding [13]. Larger lesions can also lead to airway obstruction [14]. 

**Figure 4:**
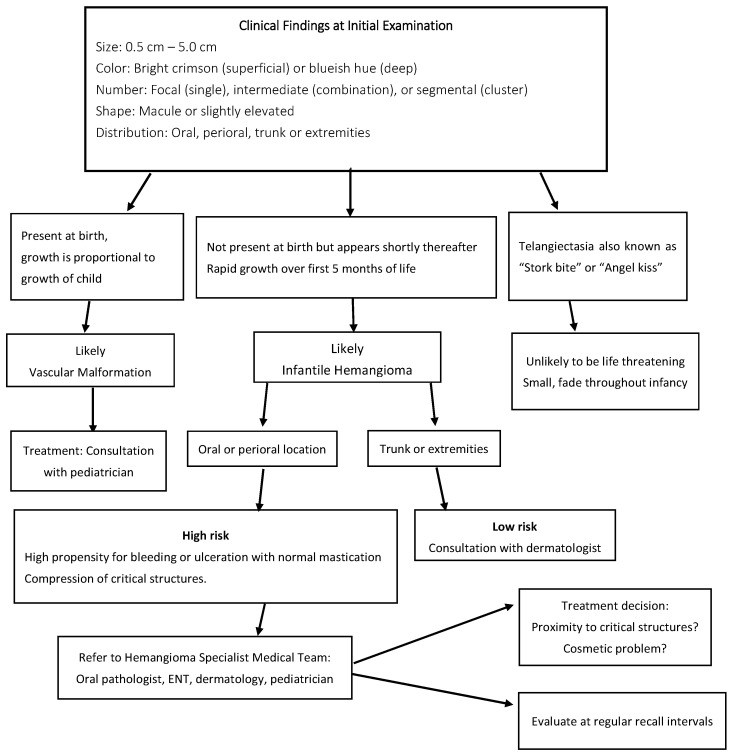
IH decision tree.

Specialized hemangioma teams are the best resource for patients with oral or perioral lesions. Under the care of the team, the first line of treatment for ulcerated or proliferating IH is oral propranolol [7]. A randomized control trial showed that 3 mg/kg per day for 6 months was an effective treatment for the lesion [7]. There was a notable greater mean reduction in hemangioma surface area as well as color intensity with treatment, with few reports of side effects. With the adoption of propranolol as the first line of treatment and with good tolerance, corticosteroid therapy has become less and less accepted as first-line therapy (See Figure 4).

## 4. Conclusions

In conclusion, although the lesions of IH are generally benign in nature, there still needs to be a proper diagnosis and understanding of the prognosis, clinical course, and treatment rationale. Smaller lesions less than 2 cm and not located on the face or in the oral cavity have much less risk associated with them. Lesions located in the oral cavity or that are larger should be referred to a specific hemangioma team for evaluation. The role of the dentist or other health care provider is to make an accurate identification of the lesion, provide guidance to the family, and provide proper referrals for expert evaluation such as an oral and maxillofacial pathologist, dermatologist, or ENT.

## Data Availability

Not applicable.
